# Integrated Impedometric Approach for Screening Natural Preservatives Against Food-Related Microorganisms

**DOI:** 10.3390/foods15142421

**Published:** 2026-07-08

**Authors:** Luca Zignego, Olimpia Pitirollo, Annalisa Ricci, Margherita Lanzi, Antonella Cavazza, Luca Bettera, Monica Gatti, Elena Bancalari

**Affiliations:** 1 Department of Food and Drug, University of Parma, Parco Area Delle Scienze 49/A, 43124 Parma, Italy; luca.zignego@unipr.it (L.Z.); olimpia.pitirollo@unipr.it (O.P.); annalisa.ricci@unipr.it (A.R.); luca.bettera@unipr.it (L.B.); monica.gatti@unipr.it (M.G.); 2 Department of Chemical Science, Life Science and Environmental Sustainability, University of Parma, Parco Area Delle Scienze 17/A, 43124 Parma, Italy; margherita.lanzi@unipr.it (M.L.); antonella.cavazza@unipr.it (A.C.); 3 Interdepartmental Centre for Packaging—CIPACK, University of Parma, Parco Area Delle Scienze, 43124 Parma, Italy

**Keywords:** multifunctional natural preservatives, impedometric analysis, antimicrobial assessment, antioxidant evaluation, microbial food spoilage

## Abstract

The growing demand for natural and sustainable preservatives has renewed interest in essential oils (EOs) because of their potential antimicrobial and antioxidant activities. In this study, lemon, lavender, and oregano EOs, together with tomato and artichoke extracts, were evaluated against foodborne and spoilage microorganisms using an impedometric approach. Three bacterial strains (*Listeria innocua*, *Escherichia coli*, and *Pseudomonas fluorescens*) and two yeast strains (*Zygosaccharomyces bailii* and *Kluyveromyces marxianus*) were tested. Tomato and artichoke extracts showed no antimicrobial activity under the tested conditions, with MIC values higher than 66.67 µL/mL. Among the EOs, oregano EO showed the strongest antimicrobial properties, with both bacteriostatic and bactericidal effects, even at low concentrations for all tested strains. In contrast, lavender EO was the most effective against yeast, with MIC and MFC values of 0.75 µL/mL for both species. Antioxidant activity, assessed via DPPH and Oxitest assays, was highest for oregano EO, which showed a 95% total antioxidant capacity and an induction period of 1224 min. Overall, the results support the use of impedance-based methods as a rapid and informative tool for screening natural preservatives and identify oregano EO as the most promising multifunctional candidate, combining strong antibacterial and antioxidant properties.

## 1. Introduction

Ensuring food safety and extending the shelf life of food remain major challenges for the food industry, as both pathogenic and spoilage microorganisms can compromise product quality, stability, and consumer health. In addition to well-known foodborne pathogens, spoilage bacteria and yeasts are responsible for important economic losses and reduced product acceptability. In this context, the growing demand for clean-label and sustainable foods has stimulated increasing interest in natural preservatives that are able to control microbial growth during a food’s shelf-life while preserving product quality [1]. Although conventional preservatives remain effective and widely used, there is increasing interest in identifying natural alternatives or complementary preservation strategies that may reduce the use of synthetic additives while maintaining food safety and stability.

Among the most promising candidates, essential oils (EOs) and plant extracts (PEs) have attracted considerable attention because of their antimicrobial and antioxidant potential. Essential oils are rich in bioactive compounds, including terpenes and phenolic constituents, which can exert antimicrobial effects through membrane disruption, metabolic interference, and inhibition of key cellular functions [2,3,4]. In addition, both EOs and PEs may contribute to food preservation through antioxidant activity, thus offering a multifunctional potential that is particularly attractive for food applications [5]. Beyond food preservation, plant-derived bioactive compounds, including essential oils and plant extracts, have also attracted interest in cosmetic and pharmaceutical applications because of their antimicrobial and antioxidant properties, which may contribute to product stability and protection against microbial contamination [3]. Agro-industrial byproducts have also emerged as valuable sources of bioactive compounds, in line with circular economy strategies and the valorization of food processing residues [6,7,8].

Despite their potential, the application of EOs and PEs in food systems still faces several challenges. Essential oils are characterized by poor water solubility and may require concentrations that negatively affect the sensory properties of food. In addition, the stability and effectiveness of bioactive compounds can be influenced by processing conditions, storage, and interactions with food components. These limitations highlight the need for rapid and reliable screening methods capable of identifying the most promising natural preservatives before their application in complex food matrices [2,3,5]. Therefore, EOs and PEs should not be considered as direct or universal replacements for conventional preservatives, but rather as complementary tools or partial alternatives within integrated preservation strategies, such as hurdle technology, active packaging, or formulations aimed at reducing the use of synthetic preservatives.

Currently, antimicrobial activity is still mainly evaluated using conventional end-point techniques, such as agar diffusion or dilution-based assays. Although widely applied, these methods provide limited information on the dynamics of microbial inhibition and do not directly monitor metabolic activity over time [9]. In contrast, impedometric analysis detects changes in electrical impedance associated with microbial metabolism and therefore represents a rapid, sensitive, and reproducible approach for monitoring growth and inhibition in real time [10,11]. This method was selected because it enables the evaluation of growth kinetics, lag-phase extension, and MIC, MBC, and MFC determination while reducing manual sampling and operator-dependent variabilities. Moreover, because multiple replicates and conditions can be monitored in parallel, impedometric analysis provides a large number of time-resolved data points and supports higher-throughput screening of natural antimicrobial compounds.

Although impedometric analysis has already shown promising results for the evaluation of antimicrobial activity against bacteria, its potential has been far less explored for food spoilage yeasts, particularly through indirect measurements. For instance, Bancalari et al. [10] demonstrated the usefulness of this method for determining the minimum inhibitory concentration (MIC) and minimum bactericidal concentration (MBC) of *Arthrospira platensis* extract against *Listeria innocua*, *Pseudomonas fluorescens*, and *Serratia liquefaciens*, highlighting its value as a rapid and sensitive alternative to conventional assays [10]. Recent reviews have further highlighted the potential of impedometric analysis as a rapid and versatile tool for food microbiology applications, including microbial detection, starter culture characterization, and antimicrobial screening [12]. However, its application to food spoilage yeasts, particularly through indirect impedometric measurements, remains limited. Moreover, impedance-based methods have rarely been used within an integrated screening framework combining antimicrobial and antioxidant evaluations of natural preservatives. This represents a relevant gap since spoilage yeasts such as *Zygosaccharomyces bailii* and *Kluyveromyces marxianus* play an important role in the deterioration of acidic, sugary, dairy, and fermented products.

Another limitation in current screening approaches is that antimicrobial and antioxidant properties are often assessed separately, although both functions are relevant for food preservation. A combined evaluation may help identify natural compounds with complementary or multifunctional properties, supporting a more rational selection of candidates for food applications.

Based on this background, this study aimed to investigate an integrated impedometric approach for screening the antimicrobial activity of lavender, lemon, and oregano essential oils, as well as tomato and artichoke extracts, against selected food-related bacteria and spoilage yeasts. The antimicrobial assessment was combined with an antioxidant evaluation using DPPH and Oxitest assays, with the overall aim of identifying natural compounds with multifunctional potential for food preservation and providing a rapid framework for the preliminary selection before application in a real food system.

## 2. Materials and Methods

### 2.1. Chemicals and Essential Oils

The EOs were purchased commercially, and according to the information provided by the suppliers, lemon essential oil was obtained by cold pressing of lemon peels (Gisa S.r.l, Milan, Italy), whereas lavender (FLORA, Tuscany, Italy) and oregano (Essences, Nikolovo, Bulgaria) essential oils were obtained by steam distillation.

Detailed chemical characterizations, such as GC–MS profiling, were not available for the commercial essential oils used in this study.

### 2.2. Plant Extracts

Tomato byproducts (peels and seeds) and artichoke byproducts (bracts and stems) obtained from the company Greci Industria Alimentare spa (Ravadese, Italy) were dried at 40 °C for 48 h and minced to obtain powders. The extraction of bioactive compounds from tomato and artichoke byproduct powders was performed, considering the results reported by Li et al. [13]: 1 g of each sample was extracted for 15 min at 65 °C with 96% *v*/*v* ethanol (35 mL) and then submitted to ultrasound-assisted extraction for an additional 15 min using an ultrasonic bath operating at 40 kHz and 70 W. This technology provides high yields even at short treatment times. The mixture was filtered, the supernatant collected, and the residue extracted a second time under the same conditions. The supernatants were combined (total volume 70 mL), the solvent was evaporated using a rotary evaporator (Buchi, Flawil, Svizzera), and the residue was recovered in 7 mL of EtOH. The resulting samples were filtered through a 0.45 μm PTFE filter and subjected to colorimetric assays.

### 2.3. Microbial Strains and Culture Conditions

Three bacterial strains were used in the experiments: *P. fluorescens* 5026 and *L. innocua* Lin6, both belonging to the University of Parma Culture Collection (UPCCO), and *E. coli* 11229, purchased from the American Type Culture Collection (ATCC) (Manassas, VA, USA). Two yeast strains were also used: *Z. bailii* 8766, purchased from ATCC, and *K. marxianus* 6014, belonging to UPCCO (Table 1). All bacterial strains were preserved as frozen stock cultures in Tryptic Soy Broth (TSB) (Oxoid Ltd., Basingstoke, UK) with 20% (*v*/*v*) glycerol at −80 °C and were sub-cultured twice overnight under aerobic conditions in TSB (2% *v*/*v*) at 37 °C for *L. innocua* and *E. coli* and at 30 °C for *P. fluorescens*. For the yeast strains, the same procedure was followed using Yeast Peptone Dextrose (YPD) (Oxoid Ltd., Basingstoke, UK) broth with incubation at 30 °C.

### 2.4. Evaluation of MIC and MBC Against Bacteria by Direct Impedometric Analysis

**Figure 1:**
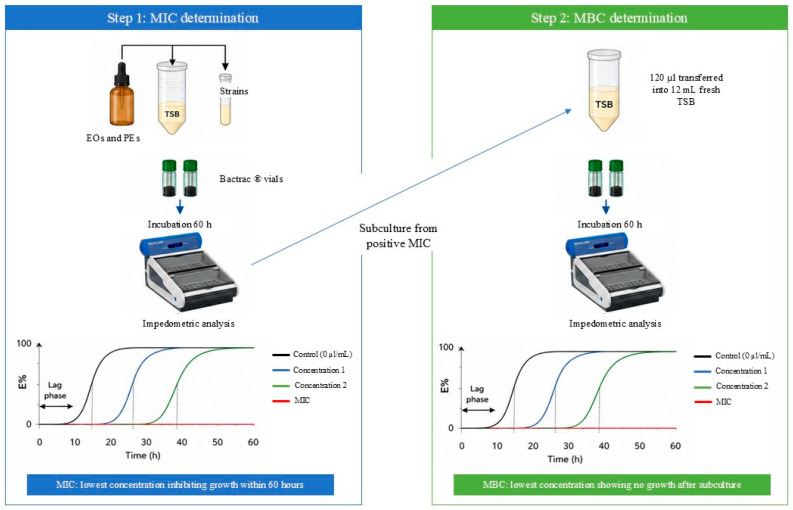
Schematic representation of the workflow used for MIC and MBC determination by direct impedometric analysis. Mic values were determined from lag phase measurements obtained during the first incubation step, whereas MBC values were assessed after subculturing cell exposed to inhibitory concentrations into fresh medium and performing a second impedometric analysis.

To establish the MIC values, 12 mL of TSB medium was individually supplemented with lemon, lavender, or oregano essential oil (EO) and tomato or artichoke extract (PE) at the following concentrations (µL/mL): 0.75, 1.55, 6.2, 12.5, 25, 33.33, 50, 66.67, and 0 (negative control without oil or extract addition). The concentration range was selected as a broad preliminary screening gradient to cover both low and high concentrations of the tested compounds and to identify concentration-dependent inhibitory responses through impedometric monitoring. EOs and PEs were directly diluted from their stock solutions into the culture medium, into which the microorganisms were subsequently inoculated. Then, 120 µL of each revitalized bacterial culture was separately added, resulting in a final concentration of 7.0 log CFU/mL. Each prepared sample was equally divided into two sterilized BacTrac 4300^®^ glass measuring cells (vials) with four electrodes (SY-LAB, Neupurkersdorf, Austria), which were incubated at the optimal growth temperature of each strain: 37 °C for *L. innocua* and *E. coli* and 30 °C for *P. fluorescens*, following the protocol reported by Bancalari et al. [10]. For MBC evaluation, after 60 h of incubation, the contents of the vials corresponding to the EO concentration that exhibited MIC activity were aseptically mixed in a 15 mL Falcon tube separately for each strain and tested compound. Then, 120 µL of each sample was used to inoculate 12 mL of fresh TSB (Figure 1). The inoculated medium was divided into two sterilized BacTrac 4300^®^ glass vials with four electrodes and incubated in the BacTrac 4300^®^ system (SYLAB, Neupurkersdorf, Austria) (Figure 1). Growth was monitored for 60 h by measuring the impedometric signal every 10 min, as previously described by Bancalari et al. [10]. The specific impedance E% value was measured and recorded every 10 min for 60 h [10,14]. Each experiment was replicated twice, and each analytical variable was measured in triplicate. The results of the impedometric measurements were analyzed, and the lag values were extrapolated following the method described by Bancalari et al. [14]. In the present study, the minimum inhibitory concentration (MIC) was defined as the lowest concentration of the tested antimicrobial able to inhibit bacterial growth. Growth inhibition was assessed using lag time values obtained from impedometric measurements. Specifically, the absence of growth was indicated by the lack of recorded lag values within 60 h.

The minimum bactericidal concentration (MBC) was determined by sub-culturing cells previously exposed to the concentrations used for MIC evaluation into fresh TSB medium. The MBC was defined as the lowest concentration at which no growth occurred in the sub-culture, i.e., when no lag values were recorded within 60 h. To evaluate the inhibitory effect, lag values obtained in the presence of different concentrations of antimicrobials were compared with those of the negative control. If the lag values were equal to the control, no inhibitory effect was detected; conversely, if the lag values increased with higher extract concentrations, an inhibitory effect on bacterial growth was observed. In the data presentation, detected lag values were expressed in hours. When a condition was not tested, N/T (not tested) was reported.

### 2.5. Evaluation of MIC and MFC Against Yeast by Indirect Impedometric Analysis

To determine the MICs and MFCs of EOs and PEs against *K. marxianus* and *Z. bailii*, the same approach and instrument used for bacteria were employed. However, instead of using BacTrac 4300^®^ glass vials with four electrodes, BacTrac 4300^®^ plastic vials were used for the indirect measurements. These vials consisted of an outer chamber, where the electrodes are located (and 2 mL of 0.1 M KOH was added), and a smaller inner vial containing the sample to be analyzed [15]. In contrast to the direct method, indirect impedance analysis monitors yeast growth through metabolic gas production, mainly CO_2_, which diffuses into the alkaline KOH solution and induces changes in its conductivity. Another difference from the direct impedance analysis used for bacteria was the use of YPD instead of TSB. In all other respects, the procedure was identical, as outlined in Figure 1. The specific impedance E% value was measured and recorded every 10 min for 30 h. Each experiment was performed in duplicate, and each analytical variable was measured in triplicate. The results of the indirect impedometric measurements were analyzed, and the lag values were extrapolated.

### 2.6. Processing of Impedometric Data for MIC and MBC Determination

The efficacy of the tested essential oils (EOs) and plant extracts (PEs) was evaluated by impedometric analysis, following the approach previously described by Bancalari et al. [10]. Lag values obtained from impedometric measurements were used to determine the minimum inhibitory concentrations (MICs) and minimum bactericidal concentrations (MBCs) against *Escherichia coli*, *Listeria innocua*, and *Pseudomonas fluorescens*.

The MIC is defined as the lowest concentration able to inhibit bacterial growth, as indicated by the absence of detectable growth within 60 h of incubation (lag time > 60 h). To determine the MBC, cells previously exposed to the concentrations tested for MIC determination were sub-cultured in fresh TSB medium. The MBC is defined as the lowest concentration at which no growth was observed after 60 h of incubation, according to Bancalari et al. [10]. For each strain, control samples without EOs or PEs (0.0 µL/mL) were included to evaluate the effect of the tested compounds on microbial growth. Lag-phase values, obtained in the presence of different concentrations of EOs or PEs, were compared with those of the untreated control.

### 2.7. DPPH Assay

The total antioxidant capacity (%TAC) of the samples (essential oils and plant extracts) was determined by UV–Vis spectrophotometry (Thermo Scientific™ Evolution™ 201/220, Milan, Italy) using the DPPH (2,2-diphenyl-1-picrylhydrazyl) assay. This assay is based on the reaction between antioxidants present in the samples and the DPPH• radical, which acts as a radical scavenger.

The PEs were used in ethanol, while the EOs were diluted in methanol at an EO/methanol ratio of 1:10. The method used for the spectrophotometric assay was reported by Pasqualone et al. [16]. Briefly, 1500 µL of DPPH• 60 µM in methanol was added to 500 µL of each sample. After 16 min of reaction, the absorbance was recorded at 517 nm using a UV–Vis spectrophotometer. At the same wavelength, but at t = 0 min, the absorbance of the DPPH• 60 µM methanolic solution (reference) was measured. The %TAC was calculated as follows:

%TAC = (Ar − As)/Ar × 100.

The analysis was performed in triplicate, and ANOVA was used for statistical analysis.

### 2.8. Oxitest

Oxidative stability was assessed using an Oxitest reactor (Velp Scientifica, Usmate, Italy) on vegetable oil, which was used as a food model and supplemented with EOs and PEs. Stability was evaluated by accelerating the oxidation process under controlled conditions of a constant temperature (90 °C) and oxygen pressure (6 bar). Sunflower oil was used as the reference sample. Ten grams of oil was analyzed in each chamber. The sunflower oil was enriched with 200 µL of each EO. To ensure a homogeneous mixture, the PE powders were infused in the model oil for 48 h before analysis. Using the Oxitest software (VELP Scientifica), it was possible to monitor the oxidative process within the two reaction chambers, graphically display the oxidation curve, and calculate the induction period (IP) as an indicator of oxidative stability.

### 2.9. Statistical Analysis

Oxidative stability and total antioxidant capacity data were processed in Microsoft Excel, and ANOVA was used for statistical analysis using Microsoft Excel for Microsoft 365. Impedometric data were analyzed using the nonparametric Kruskal–Wallis test to compare multiple independent groups when normality assumptions were not satisfied. When statistically significant differences were observed, pairwise comparisons were performed using Dunn’s post hoc test.

## 3. Results

### 3.1. Direct Impedometric Analysis for the Evaluation of Bacterial MICs and MBCs

The direct impedometric approach provided a rapid and informative evaluation of the antibacterial activity of the tested essential oils and plant extracts, highlighting clear strain- and concentration-dependent differences and enabling the identification of both bacteriostatic and bactericidal effects. Tomato and artichoke extracts did not show antibacterial activity under the tested conditions, whereas the essential oils exhibited clear strain- and concentration-dependent inhibitory effects.

Previous studies on tomato byproduct extracts have reported inconsistent antimicrobial results, ranging from activity against both Gram-positive and Gram-negative bacteria to effects mainly limited to Gram-positive species [17,18,19,20]. Evidence on artichoke byproduct extracts is more limited and mainly concerns Gram-positive bacteria and yeasts [21,22]. Unlike the tested PEs, the EOs showed variable effects depending on both concentration and strain (Table 2). All tested essential oils affected the growth of *L. innocua*, increasing its lag time compared with the control, although with different intensities depending on the oil tested. In particular, oregano essential oil inhibited *L. innocua* growth even at the lowest tested concentration (0.75 µL/mL), lavender at 6.2 µL/mL, and lemon, which was the least effective, at 12.5 µL/mL (Table 2). The MBC for lemon and lavender EOs coincided with their respective MICs, whereas the bactericidal activity of oregano EO was observed at a higher concentration (6.2 µL/mL) than the respective MIC value (0.75 µL/mL) (Table 2). MIC values comparable with those observed in the present study were reported for *L. monocytogenes* (0.039 mg/mL) by Ben Hsouna and colleagues [23]. Compared with the other EOs tested, lemon EO exhibited a lower effect, with an MBC of 12.5 µL/mL against *L. innocua*, while displaying no antibacterial activity against the other tested bacterial strains. In particular, none of the tested concentrations of lemon essential oil delayed the lag phase of *E. coli*, indicating no effect on its replication cycle. On the other hand, a slight effect was observed on the metabolism of *P. fluorescens*, whose growth was slowed down, as shown by increasing lag values with higher EO concentrations, although it was never completely inhibited (Table 2).

The results contrast with those reported in previous studies, where lemon EO showed antimicrobial activity, inhibiting the growth of various bacteria commonly found in food, such as *E. coli* and Salmonella [24]. Other studies reported different results, with MIC values ranging from 2.5 to 0.6 mg/mL and inhibitory effects against *Bacillus subtilis*, *Bacillus cereus*, *Staphylococcus aureus*, *Staphylococcus epidermidis*, *Enterococcus faecalis*, *Salmonella enterica*, *E. coli*, and *P. aeruginosa*.

The discrepancies observed between the present results and previous studies may be explained by the high variability in lemon EO composition, which depends on factors such as botanical origin, extraction method, and storage conditions. In particular, the lemon essential oil used in the present study was obtained by cold pressing, a process that preserves volatile compounds naturally present in the peel but may also co-extract non-volatile constituents, including waxes and pigments, which can affect the relative abundance of antimicrobial compounds. Cold-pressed lemon oils are often characterized by high levels of limonene, a monoterpene generally considered less antimicrobial than oxygenated compounds such as citral, which are frequently associated with stronger inhibitory activity against food-related microorganisms [2,23]. More generally, differences between the antimicrobial activity observed in the present study and values reported in the literature should be interpreted considering the intrinsic variability of essential oils, which depends on botanical origin, cultivation conditions, harvest period, extraction method, and storage conditions [8,25,26]. However, because the commercial EOs used in this study were not chemically characterized in detail, direct comparisons with literature data should be made with caution. Differences in EO composition may strongly influence antimicrobial and antioxidant performance, and the absence of a GC-MS profile prevents direct attribution of the observed effects to specific bioactive constituents. Nevertheless, it does not reduce the relevance of the study since its primary aim was not to establish composition–activity relationships but to evaluate the suitability of impedometric analysis as a rapid preliminary screening tool for natural preservatives. In this context, the results may provide useful guidance for prioritizing promising compounds for subsequent chemical characterization and validation in food systems.

Compared with lemon, lavender EO was more effective, showing an increasingly stronger delaying effect as concentrations increased, although it did not completely inhibit the growth of either *E. coli* or *P. fluorescens*. It was also more effective than lemon EO against *L. innocua*, for which both the MIC and MBC coincided at a concentration of 6.2 µL/mL (Table 2). Lavender essential oil has been reported to exert antimicrobial activity against Gram-positive and Gram-negative bacteria, as well as yeasts and molds [27]. Notably, it shows greater effects against Gram-positive bacteria such as *S. aureus* and *B. cereus*, while showing milder effects against Gram-negative strains such as *P. aeruginosa*, *E. coli*, and *Salmonella* spp. [27,28]. Other authors have studied the antimicrobial activity of lavender EO, reporting MIC values ranging from 3.0 to 0.5 mg/mL (MBC values ranging from 4 to 0.75 mg/mL) for bacteria such as *L. monocytogenes*, *E. coli*, and *P. aeruginosa* [29], while Vasileva et al. [30] reported MIC values higher than 600 µg/mL. Oregano EO showed the broadest and most consistent antibacterial activity among the tested essential oils. In particular, it inhibited *L. innocua* and *E. coli* at 0.75 µL/mL, while complete inhibition of *P. fluorescens* was achieved at 1.55 µL/mL (Table 2). Bactericidal activity was observed at higher concentrations, specifically 6.2 µL/mL for *L. innocua* and 12.5 µL/mL for both *E. coli* and *P. fluorescens*, confirming that the lethal effect required a higher dose than the inhibitory one. Overall, oregano EO was the only tested EO able to show both bacteriostatic and bactericidal activity against all bacterial strains examined, highlighting a broader antibacterial spectrum compared with lemon and lavender EOs. This finding agrees with previous studies reporting strong activity of oregano EO against a wide range of Gram-positive and Gram-negative bacteria [31,32,33]. Such effectiveness has often been associated with the presence of carvacrol, regarded as one of the main contributors to the strong antimicrobial performance of oregano EO. Overall, these findings confirm the suitability of direct impedometric analysis for the rapid screening of antibacterial activity in natural preservatives, as it enables the detection of strain-specific and concentration-dependent responses while distinguishing between inhibitory and bactericidal effects. From an application-oriented perspective, this approach can support the rational selection of essential oils according to the intended microbial target. Within this framework, oregano EO emerged as the most promising antibacterial candidate among the tested samples, owing to its consistent activity against all bacterial strains examined.

### 3.2. Indirect Impedometric Analysis for the Evaluation of Antifungal Activity

In the indirect impedance technique, electrical changes are not measured directly in the inoculated growth medium. Instead, the electrodes are placed in a separate alkaline solution, typically potassium hydroxide (KOH), while the microbial culture is contained in an inner vial. In this system, yeast growth is monitored indirectly through the production of metabolic gases, mainly CO_2_, which diffuses into the alkaline KOH solution and induces changes in its conductivity. This approach is particularly suitable for yeasts because their metabolism may interfere with the direct electrical measurements conventionally used for bacteria [34]. Although indirect impedometric analysis has already been applied for monitoring yeast growth in selected matrices, its use has mainly been limited to detection purposes rather than to the evaluation of antimicrobial activity. For example, Deak and Beuchat [15] employed this technique to detect low populations of yeasts in beverage concentrates and carbonated beverages, while Johnson et al. [35] investigated the possibility of monitoring the growth of selected microorganisms, including *Candida tropicalis* and *Zygosaccharomyces rouxii*, directly in complex food matrices. However, to the best of our knowledge, the indirect impedometric approach has not yet been specifically exploited to assess the inhibitory or fungicidal activity of essential oils and plant extracts against food spoilage yeasts. In the present study, *Z. bailii* and *K. marxianus* were selected as representative spoilage yeasts because of their relevance in the deterioration of food and beverage products. In particular, *Z. bailii* is well known for its high resistance to common preservation hurdles, whereas *K. marxianus* is frequently associated with spoilage in sugar- and dairy-related environments [36,37].

As previously observed for bacteria, tomato and artichoke PEs showed no effect on the growth of any of the tested yeast strains. As the lag values obtained for tomato and artichoke extracts were comparable to those of the control at all tested concentrations, no MIC value was detected within the concentration range tested (MIC > 66.67 µL/mL). The complete lag-phase data for plant extracts are reported in Supplementary Table S1.

On the other hand, the essential oils showed clear but distinct antifungal activity against the tested spoilage yeasts. Among the tested essential oils, lavender EO showed the strongest antifungal activity, with both MIC and MFC values of 0.75 µL/mL against *Z. bailii* and *K. marxianus* (Table 2). These results indicate a marked inhibitory and fungicidal effect against both spoilage yeasts, confirming a particularly strong antifungal profile under the experimental conditions adopted. The antifungal activity of lavender EO has previously been investigated using conventional methods against different yeasts, including *Candida* spp. and *S. cerevisiae*, with reported MIC values generally ranging from 0.25 to 0.75 mg/mL, and MFC values from 0.5 to 1 mg/mL, although no activity was observed in some cases depending on the target species [29,30]. In the present study, the fungicidal concentrations observed were among the lowest reported in the literature. Such differences may reflect variability in the EO composition related to extraction procedure, cultivation conditions, raw material, or seasonality [8,28,29,33], and may also be associated with the high sensitivity of the impedometric method [12]. The antifungal activity of lavender EO has mainly been attributed to linalool, linalyl acetate, and related constituents, which are known to affect membrane integrity and fungal physiology [33,38].

In particular, linalool, an active component of lavender EO, has been shown to inhibit fungal growth and induce morphological alterations by directly or indirectly compromising the integrity and function of the plasma membrane. Furthermore, Máté et al. [38] demonstrated that linalool modulates the fatty acid profile of fungal cells by increasing the proportion of unsaturated and polyunsaturated fatty acids [38]. Lemon EO showed a selective antifungal effect, being active against *Z. bailii* (MIC 12.5 µL/mL; MFC 33.33 µL/mL) but not against *K. marxianus*, for which no MIC value was identified despite a clear concentration-dependent extension of the lag phase (Table 2). This behavior indicates a partial inhibitory effect against *K. marxianus*, which is insufficient to completely suppress growth within the experimental conditions adopted. The antifungal activity of lemon EO has previously been reported against several yeast species, although with markedly variable inhibitory concentrations depending on the microorganism tested and the methodological approach used [39]. In this respect, the present results further support the view that lemon EO exerts highly strain-dependent antifungal activity and are in substantial agreement with the literature data reporting MIC values of around 12.5 µL/mL against *Zygosaccharomyces* spp.

Tserennadmid et al. [40] and Belletti et al. [41] investigated the effect of lemon EO on the lag phases of various yeast species, reporting inhibitory effects at different concentrations: 0.125–1 µL/mL against *G. candidum*, *P. anomala*, *S. cerevisiae*, and *S. pombe*; and 300–3000 ppm against *S. cerevisiae*, respectively. In those studies, the growth rate was defined as the change in absorbance per unit of time (1/h), and the lag phase was calculated accordingly. Compared with conventional methods such as plate counts or absorbance measurements, impedometric analysis offers the advantage of automated and continuous monitoring of microbial growth. This approach allows real-time acquisition of growth data without the need for repeated sampling, thereby facilitating a more precise evaluation of growth kinetics and lag phases. As measurements are directly recorded by the instrument in a systematic and reproducible manner, the method also reduces the analysis time and limits the variability associated with manual handling [12]. Overall, the results observed in the present study are consistent with those reported by Kunicka-Styczyńska [39], who reported a MIC of 12.5 µL/mL against *Z. bailii*.

Oregano EO was found to be effective, showing MIC and MFC values of 6.2 µL/mL for *Z. bailii* and 12.5 µL/mL for *K. marxianus* (Table 2). The efficacy of oregano EO against different fungi has also been previously studied. Chami et al. [42] reported that oregano EO at a concentration of 0.025% completely inhibited the growth of *S. cerevisiae*, while a concentration of 0.033% was required for fungicidal activity. Similarly, its activity against various Candida species was investigated, revealing growth inhibition at EO concentrations ranging from 0.06 to 0.25 mg/mL [33]. Osimani and colleagues [43] also reported strong activity of carvacrol, one of the main constituents of oregano and thyme EOs, in inhibiting *Z. rouxii* and *Z. bailii* growth. Overall, the MIC values reported in the literature generally differ from those observed in the present study. This difference may be explained by the diversity of the EOs used, as well as by differences in the yeast species and genera investigated.

### 3.3. Antioxidant Activity of Essential Oils and Plant Extracts

**Figure 2:**
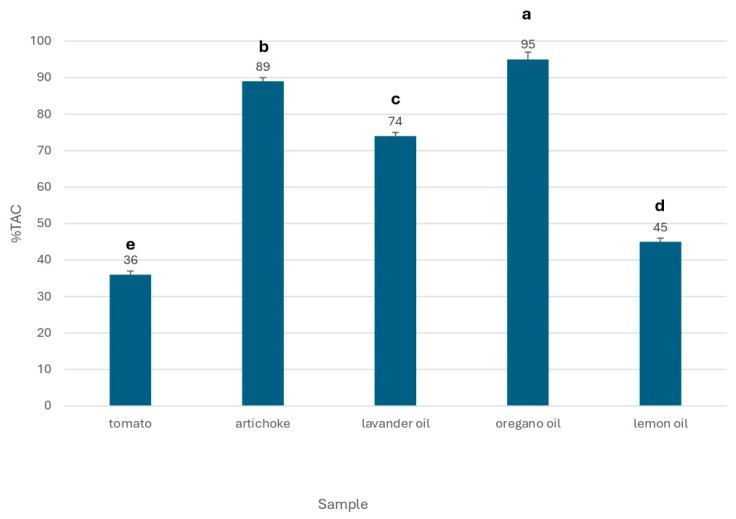
Total Antioxidant Capacity of essential oil and plant extracts. Different letters above the bars (a–e) indicate statistically significant differences among samples. Bars sharing the same letter are not significantly different.

Alongside antimicrobial activity, the antioxidant properties of the tested essential oils and plant extracts were assessed to provide a broader functional characterization of their preservative potential in food systems. The antioxidant properties of EOs and PEs were evaluated through total antioxidant capacity (%TAC) and oxidative stability assays. Total antioxidant capacity (%TAC) was measured using the DPPH• colorimetric assay, which is rapid, low cost, and characterized by high precision. As shown in Figure 2, the comparison among samples revealed notable differences.

Among the EOs, oregano showed the highest %TAC (95%), whereas lemon showed the lowest value (45%). These differences likely reflect variations in chemical composition: oregano EO contains about 65% carvacrol, whereas lavender and lemon EOs have their main active compounds in the range of 35–40%. Regarding PEs, tomato showed the lowest %TAC (36%) compared with all other samples, while artichoke extract, which is known to be rich in polyphenols such as chlorogenic acid, quercetin, and quercitrin [6], showed a %TAC of 89%, which was higher than those of lavender and lemon essential oils.

Oxidative stability, assessed by Oxitest, further highlighted relevant differences among the tested samples. The Oxitest reactor has been proposed as a tool to evaluate the direct effect on whole food products without the need for pretreatment or fat extraction [44,45]. The method is based on the acceleration of the oxidation process by applying high oxygen pressure and temperature. The Oxitest results provide the induction period (IP), which measures the time required to reach the starting point of oxidation, corresponding to a sudden change in oxygen pressure in the reactor. Compared with traditional analytical systems, Oxitest can evaluate the overall effect exerted by all active compounds on sample oxidative stability, thus providing results related to the whole system. Indeed, the contributions of possible pro-oxidants, antioxidants, and potential synergistic effects are also considered. Sunflower oil was selected to perform the Oxitest analysis because a lipid matrix is required. The effects of EOs and PEs on the IP of sunflower oil were evaluated by adding the EOs and tomato or artichoke powders to the reference oil.

**Figure 3:**
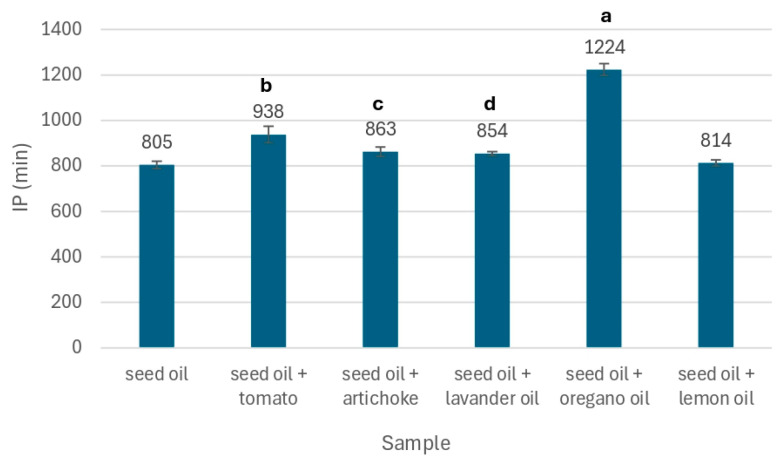
Induction Period of sunflower oil enriched with essential oil and plant extracts. Different letters above the bars (a–d) indicate statistically significant differences among samples. Bars sharing the same letter are not significantly different.

All tested extracts and oils increased the IP of the oil, indicating that they effectively enhance oxidative stability (Figure 3).

Tomato and artichoke extracts showed different behavior depending on the antioxidant assay considered. Although the artichoke extract displayed a markedly higher %TAC than tomato extract (89% vs. 36%), tomato-enriched oil exhibited a longer induction period than artichoke-enriched oil (938 ± 36.8 vs. 863 ± 20.1 min). These results suggest that radical scavenging capacity and oxidative stabilization in a lipid matrix do not necessarily coincide. This discrepancy may be related to differences in the chemical nature of the antioxidants involved and to their affinity for the oil system, with tomato byproducts possibly contributing more lipophilic compounds, such as carotenoids, which are particularly effective in the Oxitest model. Comparing the results of the DPPH• assay and the Oxitest reactor, oregano essential oil showed the highest antioxidant activity, as indicated by both %TAC and IP values, likely because of the high concentration of carvacrol. This result agrees with a previous study in which oregano EO increased the oxidative stability of olive oil [45]. A clear connection emerged between antioxidant and antimicrobial activities for oregano EO. Not only did it show the highest %TAC and IP, but it also exhibited the strongest antimicrobial effect against all tested bacterial strains. This suggests that compounds responsible for radical scavenging, such as carvacrol, may also contribute to microbial inhibition, potentially through mechanisms involving oxidative stress or disruption of microbial membranes. Conversely, PEs such as tomato and artichoke demonstrated a partial decoupling between antioxidant and antimicrobial effects. Although the artichoke extract had a higher %TAC, tomato-enriched oil displayed greater oxidative stability, and neither PE showed significant antimicrobial activity. This indicates that high antioxidant potential does not automatically predict antimicrobial efficacy, but the two properties may act in a complementary manner depending on the matrix and target microorganisms. Another point to consider is the oil’s extractive capacity, which allows it to interact with lipophilic substances that may be more abundant in tomato than in artichoke. Another possible explanation is that tomatoes are rich in lipophilic carotenoids known for their antioxidant properties [16].

**Figure 4:**
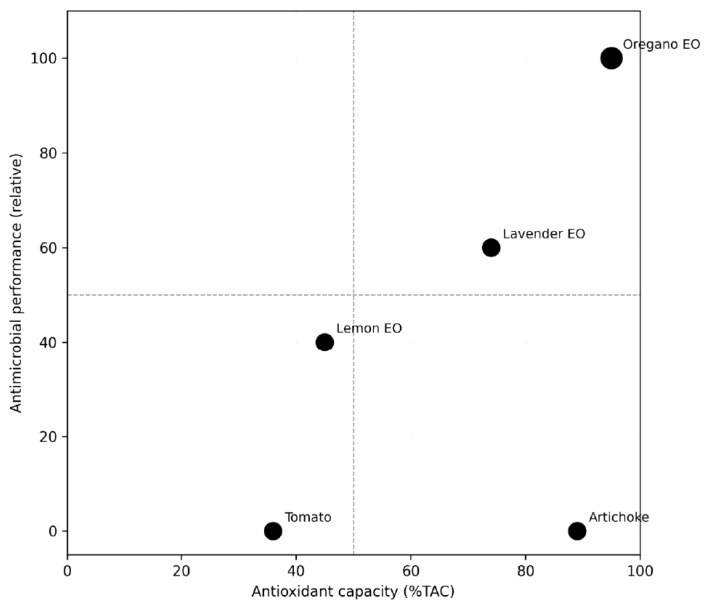
Relationship between antioxidant capacity (%TAC) and antimicrobial performance of the tested samples. Antimicrobial activity is expressed as the number of microbial strains inhibited (MIC detected) across the tested bacteria and yeasts.

Overall, these findings reinforce the importance of combining antimicrobial and antioxidant evaluations when screening natural preservatives for food applications. To provide an integrated view of the functional properties of the tested samples, antioxidant and antimicrobial data were combined in a comparative plot (Figure 4), which highlights oregano essential oil as the most effective multifunctional candidate, combining strong antimicrobial activity with high antioxidant performance, whereas tomato and artichoke extracts mainly exhibited antioxidant properties without corresponding antimicrobial effects.

Lavender essential oil displayed a relatively balanced profile, characterized by strong antimicrobial activity, with a moderate antioxidant capacity, while lemon essential oil showed an intermediate behavior, reflecting a more limited and strain-dependent antimicrobial effect. The distribution of the samples across the plot allows a rapid identification of different functional profiles, with the upper-right quadrant representing compounds with both high antioxidant and antimicrobial performance. This visualization provides a practical framework for selecting natural compounds according to the specific requirements of food systems, where both oxidative stability and microbial control are critical.

Overall, these findings demonstrate that an integrated evaluation of antioxidant and antimicrobial properties is essential to achieve a comprehensive characterization of natural compounds. This is particularly important for food applications, where the simultaneous control of oxidation and microbial growth is required to ensure product stability and safety.

## 4. Conclusions

This study demonstrated the effectiveness of impedometric analysis as a rapid and reliable tool for assessing the antimicrobial properties of essential oils (EOs) and plant extracts (PEs), enabling the determination of MIC, MBC, and MFC values and providing detailed insights into microbial growth dynamics. A clear distinction emerged between EOs and PEs in terms of biological activity. While PEs, particularly artichoke extract, exhibited high antioxidant capacity (89% TAC), they did not show antimicrobial activity under the tested conditions (MIC > 66.67 µL/mL). In contrast, oregano EO emerged as the most effective multifunctional candidate, combining strong antibacterial activity (MIC 0.75–1.55 µL/mL) with the highest antioxidant performance (95% TAC and IP of 1224 min). Lavender EO showed the strongest antifungal activity, with MIC and MFC values of 0.75 µL/mL against both yeast strains, whereas lemon EO displayed more limited and strain-dependent activity.

From an application perspective, these findings emphasize that the selection of natural preservatives should be based on an integrated evaluation of antimicrobial and antioxidant properties; this approach is particularly relevant for food systems, where both oxidative stability and microbial control must be ensured. In this context, EOs, particularly oregano EO, may represent promising candidates for natural preservation strategies, active packaging, or hurdle-technology systems aimed at enhancing product safety and shelf life.

Although this study provides a useful preliminary framework for screening natural preservatives, some experimental limitations should be acknowledged. Essential oils were added directly to the culture medium for antimicrobial testing, whereas plant extracts were recovered in ethanol after solvent evaporation. Therefore, the inclusion of a corresponding ethanol control would have further strengthened the interpretation of the results obtained with plant extracts. However, since tomato and artichoke extracts did not show antimicrobial activity under the tested conditions, the absence of this control is unlikely to have led to false positive inhibitory effects. In addition, positive antimicrobial and antioxidant controls would have allowed a more standardized comparison with reference compounds and should be included in future validation studies.

Future research should expand this work by evaluating the behavior of these compounds in real food matrices, where interactions with food components may influence their bioactivity. In addition, studies on multi-species systems and potential synergistic effects with conventional preservatives or other natural antimicrobials could support the development of more effective and application-oriented formulations requiring lower doses, thereby reducing potential sensory impacts.

## Figures and Tables

**Table 1:** Growth conditions of bacterial and yeast strains used.

ID	Source	Genus	Species	Abbreviation	Matrix	Temperature	O_2_	Medium
5026	UPCCO	*Pseudomonas*	*fluorescens*	*P. fluorescens*	Meat	30 °C	Yes	TSB
11229	ATCC	*Escherichia*	*coli*	*E. coli*	Not available	37 °C	Yes	TSB
Lin6	UPCCO	*Listeria*	*innocua*	*L. innocua*	Cheese	37 °C	Yes	TSB
8766	ATCC	*Zygosaccharomyces*	*bailii*	*Z. bailii*	Not available	30 °C	Yes	YPD
6014	UPCCO	*Kluyveromyces*	*marxianus*	*K. marxianus*	Cheese	30 °C	Yes	YPD

**Table 2:** Lag values calculated for each strain and EO concentration, used to determine MIC, MBC, and MFC values. MIC: minimum inhibitory concentration; MBC: minimum bactericidal concentration; MFC: minimum fungicidal concentration.

EO (µL/mL)	*L. innocua* *UPCCO Lis6*		*E. coli* *ATCC 11229*		*P. fluorescens* *UPCCO 5026*		*Z. bailii* *ATCC 8766*		*K. marxianus* *UPCCO 6014*	
	MIC	MBC	MIC	MBC	MIC	MBC	MIC	MFC	MIC	MFC
Lemon										
0	0.88 ± 0.01	N/T	0.00 ± 0.02	N/T	0.45 ± 0.01	N/T	0.00 ± 0.02	N/T	0.45 ± 0.01	N/T
0.75	9.74 ± 0.02	N/T	0.00 ± 0.04	N/T	0.27 ± 0.01	N/T	0.58 ± 0.34	N/T	2.61 ± 0.26	N/T
1.55	19.64 ± 0.01	N/T	0.00 ± 0.04	N/T	1.50 ± 0.01	N/T	0.97 ± 0.34	N/T	4.70 ± 0.21	N/T
6.2	46.26 ± 0.04 *	N/T	0.00 ± 0.01	N/T	1.83 ± 0.40	N/T	5.10 ± 0.42 *	N/T	5.50 ± 0.24	N/T
12.5	>60 ± 0.03 **	>60 ± 0.03	0.00 ± 0.04	N/T	3.79 ± 0.02	N/T	>60 ± 0.00 ***	12.59 ± 0.00	9.65 ± 1.12	N/T
25	>60 ± 0.03 **	>60 ± 0.03	0.00 ± 0.01	N/T	4.23 ± 0.01 *	N/T	>60 ± 0.00 ***	33.60 ± 0.00	11.87 ± 0.03 *	N/T
33.33	>60 ± 0.02 **	>60 ± 0.02	0.00 ± 0.01	N/T	3.87 ± 0.24	N/T	>60 ± 0.00	>60 ± 0.00	15.26 ± 0.99 *	N/T
50	>60 ± 0.04 **	>60 ± 0.04	0.00 ± 0.01	N/T	5.17 ± 0.42 **	N/T	>60 ± 0.00	>60 ± 0.00	22.41 ± 0.21	N/T
66.67	>60 ± 0.01 **	>60 ± 0.01	0.00 ± 0.04	N/T	5.83 ± 0.12 **	N/T	>60 ± 0.00 ***	>60 ± 0.00	28.24 ± 0.24	N/T
Lavender										
0	0.88 ± 0.01	N/T	0.00 ± 0.02	N/T	0.45 ± 0.01	N/T	0.00 ± 0.02	N/T	0.45 ± 0.01	N/T
0.75	19.47 ± 0.01	N/T	0.11 ± 0.02	N/T	0.53 ± 0.50	N/T	>60 ± 0.00	>60 ± 0.00	>60 ± 0.00	>60 ± 0.00
1.55	33.98 ± 0.01	N/T	2.36 ± 0.02	N/T	0.53 ± 0.50	N/T	>60 ± 0.00	>60 ± 0.00	>60 ± 0.00	>60 ± 0.00
6.2	>60 ± 0.02 **	>60 ± 0.02	6.51 ± 0.04 *	N/T	1.30 ± 0.10	N/T	>60 ± 0.00	N/T	>60 ± 0.00	N/T
12.5	>60 ± 0.02 **	>60 ± 0.02	7.58 ± 0.04	N/T	1.80 ± 0.01	N/T	N/T	N/T	N/T	N/T
25	>60 ± 0.04 **	>60 ± 0.04	9.63 ± 0.30 **	N/T	14.88 ± 0.30	N/T	N/T	N/T	N/T	N/T
33.33	>60 ± 0.04 **	>60 ± 0.04	8.52 ± 0.02	N/T	14.08 ± 0.02	N/T	N/T	N/T	N/T	N/T
50	>60 ± 0.01 **	>60 ± 0.01	8.55 ± 0.01 **	N/T	15.28 ± 0.01 *	N/T	N/T	N/T	N/T	N/T
66.67	>60 ± 0.01 **	>60 ± 0.01	9.57 ± 0.02 **	N/T	15.50 ± 0.02 **	N/T	N/T	N/T	N/T	N/T
Oregano										
0	0.88 ± 0.01	N/T	0.00 ± 0.02	N/T	0.45 ± 0.01	N/T	0.00 ± 0.02	N/T	0.45 ± 0.01	N/T
0.75	>60 ± 0.01	N/T	24.15 ± 0.04	N/T	>60 ± 0.00	N/T	1.34 ± 0.01	N/T	2.31 ± 0.03	N/T
1.55	>60 ± 0.02	16.50 ± 0.02	>60 ± 0.00	N/T	>60 ± 0.03	13.35 ± 0.17	5.81 ± 0.55	N/T	5.92 ± 0.44	N/T
6.2	>60 ± 0.03	>60 ± 0.03	>60 ± 0.00	N/T	>60 ± 0.03	>60 ± 0.00	>60 ± 0.00 ***	>60 ± 0.00	27.63 ± 1.65	N/T
12.5	>60 ± 0.01	>60 ± 0.01	>60 ± 0.00	>60 ± 0.03	>60 ± 0.01	>60 ± 0.00	>60 ± 0.00 ***	>60 ± 0.00	>60 ± 0.00 ***	>60 ± 0.00
25	>60 ± 0.01	>60 ± 0.01	>60 ± 0.00	>60 ± 0.03	>60 ± 0.04	>60 ± 0.00	>60 ± 0.00 ***	N/T	>60 ± 0.00 ***	>60 ± 0.00
33.33	N/T	N/T	N/T	>60 ± 0.02	N/T	N/T	N/T	N/T	N/T	N/T
50	N/T	N/T	N/T	>60 ± 0.04	N/T	N/T	N/T	N/T	N/T	N/T
66.67	N/T	N/T	N/T	>60 ± 0.01	N/T	N/T	N/T	N/T	N/T	N/T

N/T: not tested. Statistical significance was assessed using the Kruskal–Wallis test followed by Dunn’s multiple comparisons test. Asterisks indicate significant differences compared with the control (* *p* < 0.05; ** *p* < 0.01; *** *p* < 0.001). Vertical comparisons were performed between the control and the different concentrations for each microorganism and essential oil.

## Data Availability

The original contributions presented in this study are included in the article/Supplementary Materials. Further inquiries can be directed to the corresponding author.

## Supplementary Materials

The following supporting information can be downloaded at: https://www.mdpi.com/article/10.3390/foods15142421/s1, Table S1. Lag values (h) obtained for each strain at the tested concentrations of tomato and artichoke extracts, supporting MIC values > 66.67 µL/mL.
