# Supplementary material for: Integrated Impedometric Approach for Screening Natural Preservatives Against Food-Related Microorganisms
Source: Foods. 2026 Jul 8;15(14):2421. doi: 10.3390/foods15142421 (PMC13409732; doi:10.3390/foods15142421)
Supplement: Supplementary file 1 [file foods-15-02421-s001.zip › foods-4375129-supplementary.pdf]

Table S1. Lag values (h) obtained for each strain at the tested concentrations of tomato and artichoke extracts, supporting MIC values >66.67 µL/mL.

**PEs (µL/mL)**	*L. innocua* *UPCCO Lis6*		*E. coli* *ATCC 11229*		*P. fluorescens* *UPCCO 5026*		*Z. bailii* *ATCC 8766*		*K. marxianus* *UPCCO 6014*	
	MIC	MBC	MIC	MBC	MIC	MBC	MIC	MFC	MIC	MFC
Tomato extracts										
0	0.82±0.05	N/T	0.13±0.04	N/T	0.39±0.05	N/T	0.12±0.03	N/T	0.42±0.05	N/T
0.75	0.88±0.04	N/T	0.00±0.01	N/T	0.46±0.04	N/T	0.08±0.04	N/T	0.37±0.06	N/T
1.55	0.91±0.06	N/T	0.00±0.03	N/T	0.41±0.06	N/T	0.00±0.00	N/T	0.48±0.04	N/T
6.2	0.84±0.05	N/T	0.06±0.04	N/T	0.44±0.05	N/T	0.08±0.03	N/T	0.40±0.07	N/T
12.5	0.89±0.07	N/T	0.08±0.05	N/T	0.37±0.07	N/T	0.05±0.04	N/T	0.46±0.05	N/T
25	0.81±0.04	N/T	0.10±0.03	N/T	0.48±0.04	N/T	0.11±0.05	N/T	0.39±0.06	N/T
33.33	0.87±0.06	N/T	0.00±0.04	N/T	0.43±0.06	N/T	0.09±0.04	N/T	0.45±0.04	N/T
50	0.90±0.05	N/T	0.09±0.05	N/T	0.40±0.05	N/T	0.12±0.03	N/T	0.49±0.05	N/T
66.67	0.85±0.07	N/T	0.11±0.04	N/T	0.47±0.06	N/T	0.00±0.00	N/T	0.41±0.06	N/T
Artichoke extracts										
0	0.90±0.04	N/T	0.00±0.00	N/T	0.42±0.05	N/T	0.00±0.04	N/T	0.38±0.06	N/T
0.75	0.83±0.06	N/T	0.08±0.05	N/T	0.47±0.04	N/T	0.10±0.03	N/T	0.45±0.05	N/T
1.55	0.88±0.05	N/T	0.10±0.04	N/T	0.39±0.07	N/T	0.08±0.04	N/T	0.41±0.06	N/T
6.2	0.79±0.07	N/T	0.13±0.03	N/T	0.45±0.05	N/T	0.00±0.00	N/T	0.49±0.04	N/T
12.5	0.92±0.04	N/T	0.11±0.05	N/T	0.40±0.06	N/T	0.09±0.03	N/T	0.43±0.05	N/T
25	0.85±0.06	N/T	0.09±0.04	N/T	0.48±0.04	N/T	0.06±0.04	N/T	0.37±0.07	N/T
33.33	0.89±0.05	N/T	0.00±0.00	N/T	0.41±0.05	N/T	0.11±0.05	N/T	0.46±0.04	N/T
50	0.82±0.08	N/T	0.08±0.03	N/T	0.44±0.07	N/T	0.10±0.04	N/T	0.40±0.06	N/T
66.67	0.87±0.05	N/T	0.010±0.04	N/T	0.38±0.06	N/T	0.05±0.03	N/T	0.47±0.05	N/T

N/T: not tested.

Statistical significance was assessed using the Kruskal-Wallis test followed by Dunn's multiple comparisons test. Asterisks indicate significant differences compared with the control (* $p < 0.05$; ** $p < 0.01$; *** $p < 0.001$; **** $p < 0.0001$). Vertical comparisons were performed between the control and the different concentrations for each microorganism and essential oil.
